# Immunoglobulin superfamily genes are novel prognostic biomarkers for breast cancer

**DOI:** 10.18632/oncotarget.13683

**Published:** 2016-11-29

**Authors:** Yue Li, Maoni Guo, Zhenkun Fu, Peng Wang, Yan Zhang, Yue Gao, Ming Yue, Shangwei Ning, Dianjun Li

**Affiliations:** ^1^ Department of Medical Oncology, Harbin Medical University Cancer Hospital, Harbin, 150081, China; ^2^ College of Bioinformatics Science and Technology, Harbin Medical University, Harbin, 150081, China; ^3^ Department of Immunology, Harbin Medical University, Harbin, 150081, China

**Keywords:** immunoglobulin superfamily, breast cancer, network module, topology feature, prognostic biomarker

## Abstract

Breast cancer progression is associated with dysregulated expression of the immunoglobulin superfamily (IgSF) genes that are involved in cell-cell recognition, binding and adhesion. Despite widespread evidence that many IgSF genes could serve as effective biomarkers, this potential has not been realized because the studies have focused mostly on individual genes and not the entire network. To gain a global perspective of the IgSF-related biomarkers, we constructed an IgSF-directed neighbor network (IDNN) and an IgSF-directed driver network (IDDN) by integrating multiple levels of data, including IgSF genes, breast cancer driver genes, protein-protein interaction (PPI) networks and gene expression profiling data. Our study shows that IgSF genes in the PPI network have important topological features related to cancer. Most IgSF genes are either cancer driver genes themselves or associated with them. We also identified a 21-gene IgSF network module with enriched mutations that are associated with overall survival based on 450 breast cancer patient samples extracted from The Cancer Genome Atlas (TCGA) and multiple independent microarray validation datasets. These results highlight the potential of IgSF genes as novel diagnostic, prognostic and therapeutic targets for breast cancer.

## INTRODUCTION

Breast cancer is the leading cause of cancer death among women worldwide. In Chinese women, breast cancer is the most prevalent form of cancer with more than 1.6 million people diagnosed and 1.2 million people dying every year. The most common type of breast cancer is invasive ductal carcinoma (IDC) that can spread from the ducts or the lobules to the surrounding tissue. Prognostic biomarkers are useful to choose the appropriate treatment for IDC, and they significantly affect the process of cancer therapy [1–3]. Studies have shown that the genetic diversity in breast cancer impacts response to treatment and patient outcomes. This is exemplified by the estrogen receptor negative (ER−) and positive (ER+) subtypes that have different prognostic gene signatures and responses to treatment [4]. Therefore, there is scope to identify novel signatures that can enhance predicting the prognostic and clinical behavior.

Gene expression of many IgSF members is altered in breast cancer, and hence, they are promising candidates as prognostic biomarkers. ALCAM (CD166) is a potential breast cancer biomarker and a therapeutic target due to its role in induction of programmed cell death, apoptosis and autophagy in breast cancer [5]. Down regulation of CXCR4 inhibits cell migration in breast cancer cells [6]. The expression of MUC18 (CD146) promotes the progression of human breast cancer cells by increasing their motility, invasiveness and tumorigenesis [7]. L1CAM is potentially an early diagnostic biomarker in breast cancer progression as it promotes cell adhesion and migration *in vitro* [8]. Although these findings demonstrate the important role of IgSF members in breast cancer progression and metastasis, these studies focus on one or a few IgSF members analyzed in either cell lines or in limited patient samples and therefore do not present a global perspective of the entire immunoglobulin superfamily.

Current advances in cancer biology and genomic methods have generated large-scale gene expression profiling datasets (such as TCGA) and other OMICs and provide an opportunity to study the entire network of IgSF genes as well as identify novel biomarkers for breast cancer. Previously, Li and others used cancer gene microarray and network data to develop a network-based method for cancer prognostic biomarker identification [9]. Similarly, Chuang and others used a protein-network-based approach using data from protein interaction databases to identify markers as sub networks [10]. These studies demonstrate that integrating gene expression and protein-protein interaction data can improve prediction performance in network biomarker identification.

Our aim was to study the role of the IgSF network in breast cancer and test its diagnostic, prognostic and therapeutic potential. Towards this goal we constructed an IgSF-directed neighbor network (IDNN) and an IgSF-directed driver network (IDDN) to address the role of the IgSF network in breast cancer. We identified IgSF genes in the PPI network with hub topological features connected to breast cancer. We also identified a 21-gene module from the IDDN network that was associated with the overall survival of breast cancer patients. This module included several key IgSF and breast cancer driver genes with enriched mutations, demonstrating the functional significance of the IgSF genes to predict breast cancer. Our findings highlight the novel role of the IgSF-directed network in breast cancer. It also highlights their potential for biomarker-guided development of preclinical and clinical therapeutic modalities.

## RESULTS

### IgSF genes play a crucial role in breast cancer

**Figure 1:**
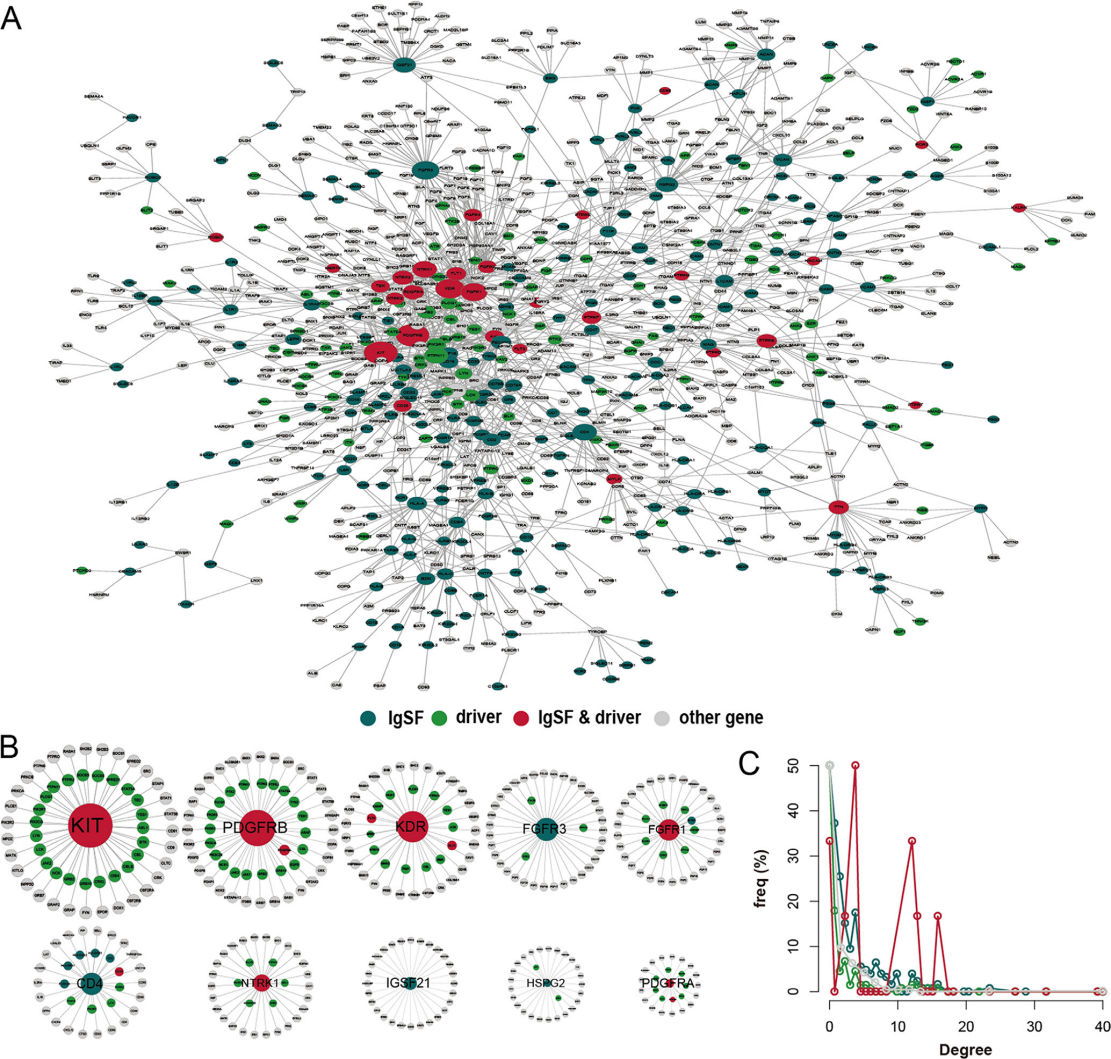
The properties of the IgSF-directed neighbor network (IDNN) (**A**) The global IDNN. (**B**) Top 10 IgSF genes ranked by gene degree (KIT, PDGFRB, KDR, FGFR3, FGFR1, CD4, NTRK1, IGSF21, HSPG2, and PDGFRA). (**C**) The degree distribution of the nodes in IDNN.

We constructed an IgSF-directed neighbor network (IDNN) that had 1050 nodes including 283 IgSF genes and their 767 neighbors from the PPI network (Figure 1A, Supplementary Table S1). We found 33 IgSF genes (KIT, PDGFRB, KDR, FGFR1, CD28, NTRK1, etc.) that were themselves breast cancer driver genes as well as 250 IgSF genes (including PTPN11, LCK, GRB2, ABL1, APP, etc. Supplementary Table S2) that associated with the breast cancer driver genes. The top 10 IgSF genes (KIT, PDGFRB, KDR, FGFR3, FGFR1, CD4, NTRK1, IGSF21, HSPG2, and PDGFRA) had a direct connection in the network, indicating that IgSF genes play hub roles in the sub-networks (Figure 1B). We also found a greater degree of genes (mean = 16.76) that were both IgSF and breast cancer driver genes in the IDNN suggesting a intricate link between the IgSF genes and breast cancer (Figure 1C, Supplementary Figure S1). Also, enrichment of IgSF cancer driver genes in specific modules suggests that the IgSF driver genes may play consequential roles in special biological modules (Supplementary Figure S2, Supplementary Table S3).

Notably, six of the top 10 IgSF genes in the PPI network were well-known breast cancer driver genes, including KIT, whose high expression occurs infrequently in breast cancer [11]. In the triple negative breast cancer, immunohistochemical expression of C-kit and mutations of PDGFRA are frequent suggesting that they are good candidates for molecular targeted therapy [12]. Our findings also suggest that CD4^+^ follicular helper T cells (Tfh) may be prognostic indicators as they are found in the breast tumors [13]. Furthermore, down-regulation of KDR expression induces apoptosis in breast cancer cells [14]. Also, FGFR1 activity is required for the survival of an FGFR1-amplified breast cancer cell line [15]. Among the IgSF neighbor genes, ABL1 and PDGFR are well known breast cancer driver genes that promote acquired resistance to aromatase inhibitor (AI) therapy in ER+ breast cancers [16]. The activation of the PDGFR and ABL1 pathways is associated with long-term estrogen deprivation in MCF7 breast cancer cells and decreased anti-proliferative response to AI treatment in primary ER-positive breast carcinomas.

### IgSF genes directly interact with breast cancer driver genes

**Figure 2:**
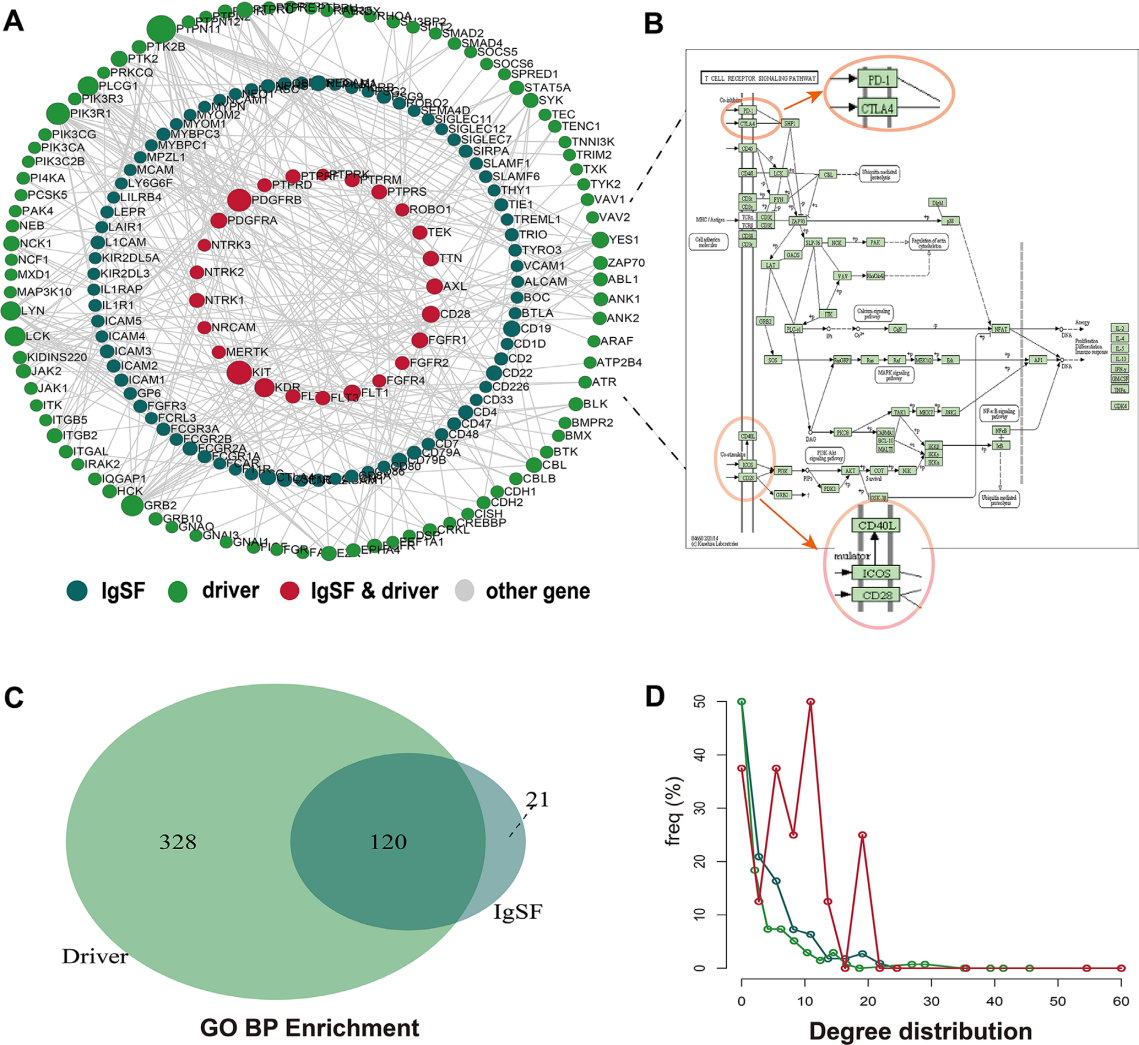
The properties of the IgSF-directed driver network (IDDN) (**A**) The global IDDN and the size of the sphere represent the degree of the gene. (**B**) The functional sub-pathway of the T cell receptor signaling pathway (**C**) The overlapping GO terms between IgSF and breast cancer driver genes. (**D**) The degree distribution of nodes in IDDN.

To further explore the relationship between IgSF and breast cancer driver genes, we constructed a network called IgSF-directed driver network (IDDN). This included IgSF genes and breast cancer driver genes that were extracted from the IDNN (Figure 2A, Supplementary Table S4). The IDDN contained 253 genes, of which 103 were IgSF and 121 were breast cancer driver genes. Among these, 29 of the IgSF genes were also breast cancer genes. Compared to the nodes in IDNN, the nodes in the IDDN had higher degrees, betweenness centrality and closeness centrality (avg. 3.440 vs. 3.220 for degrees, *p* = 0.001, Supplementary Figure S3A; avg. 0.020 vs. 0.012 for betweenness centrality, *p* = 2.74e-07, Supplementary Figure S3B; avg. 0.242 vs. 0.213 for closeness centrality, *p* = 6.78e-09, Supplementary Figure S3C; Wilcoxon rank sum test). This suggested that the IDDN obtained from IDNN was closer in structure and played a crucial role in the biogenesis of breast cancer.

To explore the biological functions of these genes, we conducted pathway analysis using the Subpathway Miner [17]. The T cell receptor signaling pathway emerged as the most significant pathway from this analysis with several of the IgSF genes located in key positions, such as CBL, LCK, ZAP70, FYN, CD28, PD-1, CTLA4, ICOS, and CD4/8 (Figure 2B). Since the expression of CBL can inhibit LCK and ZAP70 gene expression, CBL gene may play a crucial role in the pathway [18].

We then performed a GO enrichment analysis using IgSF and breast cancer driver genes. Some common GO terms that we found associated with breast cancer included cell surface receptor-linked signal transduction, immune response, regulation of cell proliferation, regulation of cell activation, regulation of T cell activation, regulation of T cell differentiation, and negative regulation of the immune system process. Significance of this analysis is exemplified by the fact that mutations of BTLA, CD28, CD4, and CD8A genes in the GO term cell surface receptor-linked signal transduction contribute to sporadic breast cancer risk. More importantly, we found overlapping GO terms between the IgSF genes and the breast cancer driver genes, including phosphate metabolic process, protein amino acid phosphorylation, enzyme linked receptor protein signaling pathway, transmembrane receptor protein tyrosine kinase signaling pathway, cell surface receptor-linked signal transduction, and regulation of cell proliferation (Figure 2C). This indicated a close connection between IgSF genes and breast cancer. Additionally, the degree of the common genes of IgSF and the breast cancer driver genes derived from the IDDN was also greatest (Figure 2D).

### IgSF genes associate with driver genes in functional modules

**Figure 3:**
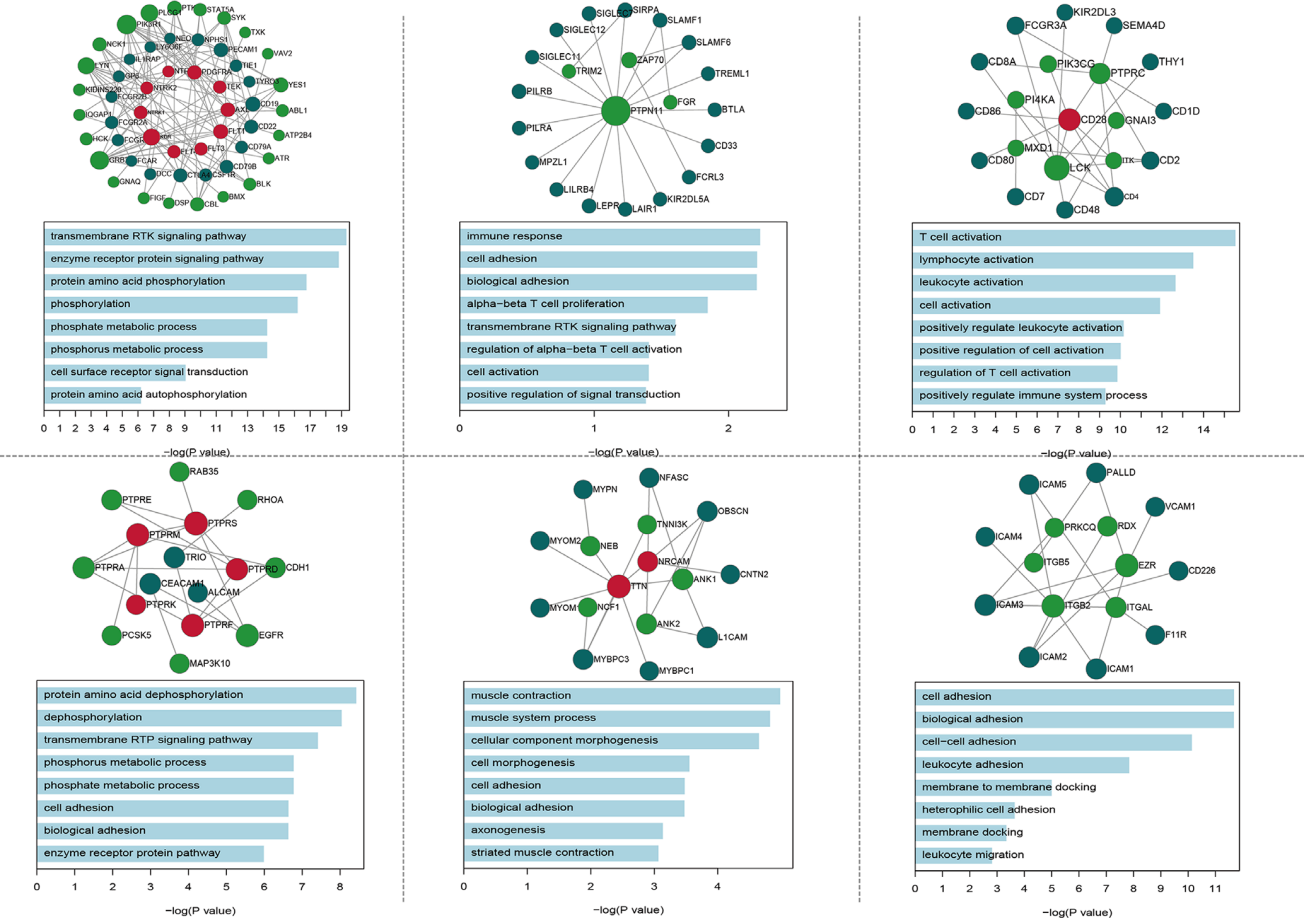
The GO BP terms of six significantly functional modules

To understand the communication between the IgSF and the breast cancer driver genes, we performed a module analysis in the IDDN. We found six significant IgSF-associated modules in which IgSF and breast cancer driver genes were closely associated (Figure 3). We conducted a GO terms enrichment analysis of the module genes using DAVID [19] and identified the biological process terms that enriched the module genes using a cutoff of FDR < 0.05 (Supplementary Table S5). The top-ranked GO terms included immune response, cell adhesion, biological adhesion, regulation of alpha-beta T cell proliferation, transmembrane receptor protein tyrosine kinase signaling pathway, and T cell activation. The GO term cell adhesion enriched by the second and sixth module genes is a very important biological process of the IgSF genes (VCAM1, ICAM1, F11R, ITGAL, EZR, ICAM4, ICAM5, ICAM2, ICAM3, ITGB5, ITGB2, and CD226) that participate in the immune response. Among these, the expression of members of the ICAM family regulates tumorigenesis and are potential diagnostic biomarkers and therapeutic targets for breast cancer [20]. Moreover, both the first and fourth modules enriched the common GO term phosphate metabolic process, which is differentially regulated in breast cancer [21].

### IgSF-related modules are enriched with cancer mutations

Since genetic mutations can cause cancer, we analyzed if IgSF genes in our modules are mutated. Previously, the IgSF genes in the module that includes well-known breast cancer genes have been shown to be mutated. Some SNPs in six immunological genes, BTLA, ITGAL, CTLA4, ICOS, PDCD1, and VTCN1 were reported as breast cancer risk mutations in previous studies [22–27] (Supplementary Table S6). We performed gene mutation enrichment analysis and evaluated if these module genes were enriched in the top 15% of mutated genes in breast cancer. We identified three module genes that overlapped with the top 15% mutated genes in breast cancer (*p* = 0.001 for module 1, Supplementary Figure S4A; *p* = 0.001 for module 4, Supplementary Figure S4B; *p* = 1.28e-07 for module 5, Supplementary Figure S4C; Hypergeometric Test).

**Figure 4:**
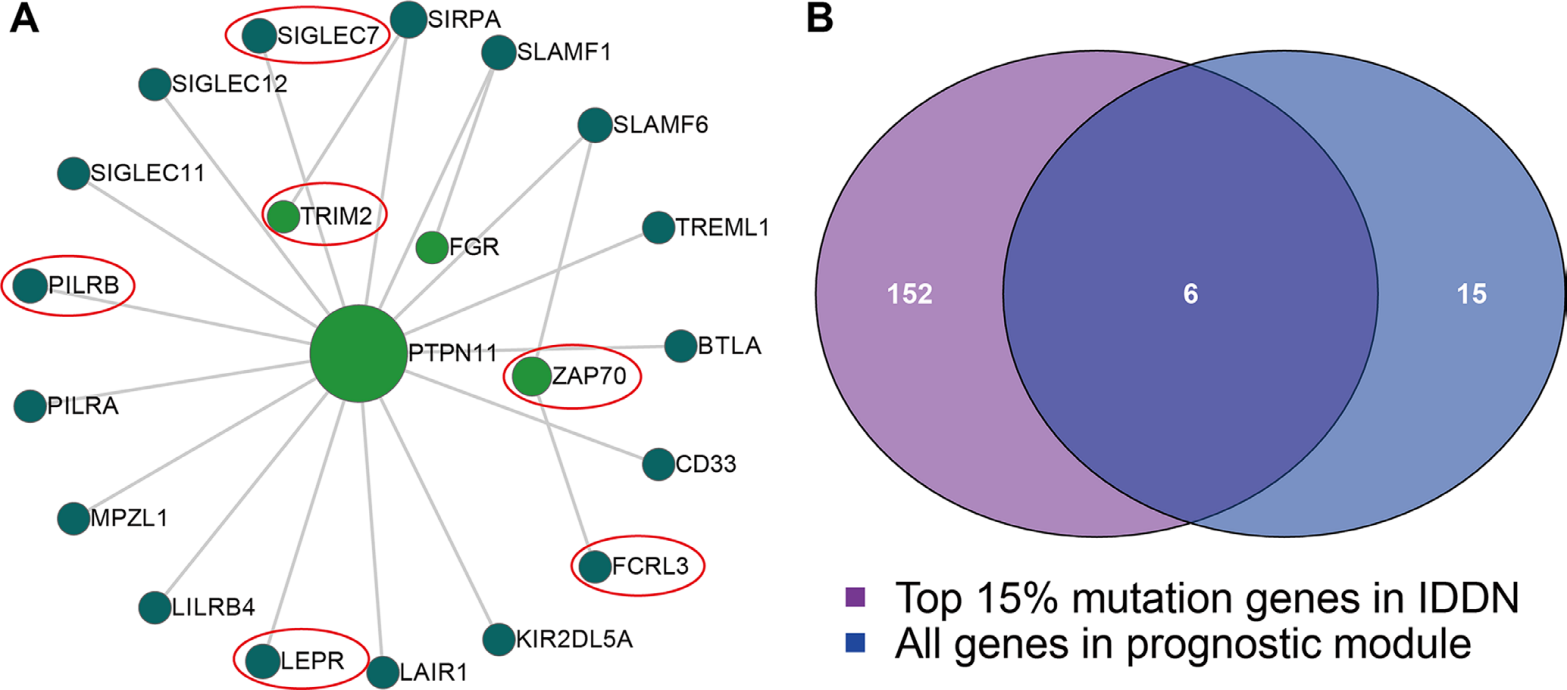
The module with mutated IgSF genes (**A**) The module includes 21 genes, with the size of the sphere depicting the degree of the gene and the red circles showing the six common mutated genes found in this module. (**B**) A Venn plot between the IgSF and the mutated genes.

Next, we identified the somatic mutations of the genes in the IDDN using TCGA breast cancer somatic mutation data and determined if they overlapped with the top 15% mutated genes in the IDDN using hypergeometric test. We found that the second module with 21 genes was enriched in mutated genes (Figure 4A, P < 0.001). Four of the six common genes (PTPN11, TRIM2, FGR and ZAP70) were IgSF genes (Figure 4B). PTPN11, a HER2-inhibition up-regulated PTP (protein tyrosine and dual-specificity phosphatase) transduces positive signals and is an oncogene [28]. TRIM2 is a diagnostically significant and conserved element of the SOX10 signature in BBC (breast basal-like carcinomas) cell lines [29]. FGR and ZAP70 have diagnostic and therapeutic potential due to their relationship with breast cancer development and progression [30, 31].

### IgSF-mutated module has prognostic potential

**Figure 5:**
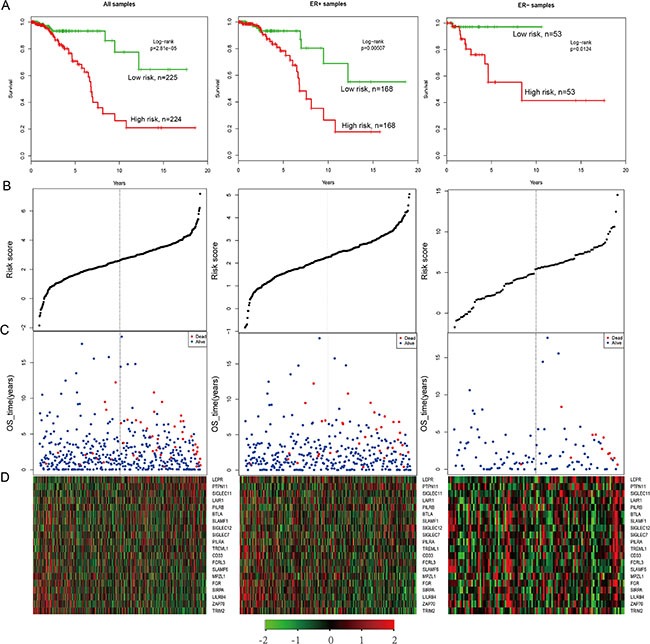
The survival analysis of the IgSF-mutated module (**A**) The Kaplan–Meier curve for the overall survival of two patient groups with high and low risk scores in the TCGA patient set (*n* = 450), ER+ samples (*N* = 337) and ER– samples (*N* = 106). The difference between the two curves was evaluated by a two-sided log-rank test. (**B**) The gene-based risk score distribution of the 20 genes (**C**) The gene-based patient survival status of the 20 genes. (**D**) The heat map depicting expression profiles of the 20 genes. The black dotted line represents the cutoff value of the risk score derived from the corresponding set that separates patients into high- and low-risk groups.

In the TCGA gene expression data, only 20 genes (BTLA, CD33, FCRL3, FGR, LAIR1, LEPR, LILRB4, MPZL1, PILRA, PILRB, PTPN11, SIGLEC11, SIGLEC12, SIGLEC7, SIRPA, SLAMF1, SLAMF6, TREML1, TRIM2, and ZAP70) had expression values. We created a risk-score formula according to the expression of these 20 genes to generate OS (overall survival) prediction (see the Material and Methods section). The Cox regression coefficients of the total samples, ER+ samples and ER– samples are as listed in Supplementary Table S7. Using the median risk score of the test series as the cutoff point, we calculated the risk scores for the 20 genes for each patient and then ranked the patients according to their risk score. The patients grouped into a high-risk (*N* = 225) or a low-risk (*N* = 225) category by using the median risk score of the test series as the cutoff point. Patients in the high-risk group had significantly shorter median OS than those in the low-risk group (Figure 5A, HR = 3.82, *P* = 2.81e-05). In addition, patients with high risk in the ER+ series (HR = 2.78, *P* = 0.00507) as well as the ER– series (HR = 8.77, *P* = 0.0124) had significantly shorter median OS than those in the low-risk group (Figure 5A). Based on the distribution of gene risk score, the survival status and the gene expression signature of the breast cancer patients, patients with high risk scores expressed higher levels in the ten risk genes, whereas patients with low risk scores expressed higher levels in the remaining ten protective genes (Figure 5B–5D).

### Validating the prognostic potential of the IgSF module in independent patients

**Figure 6:**
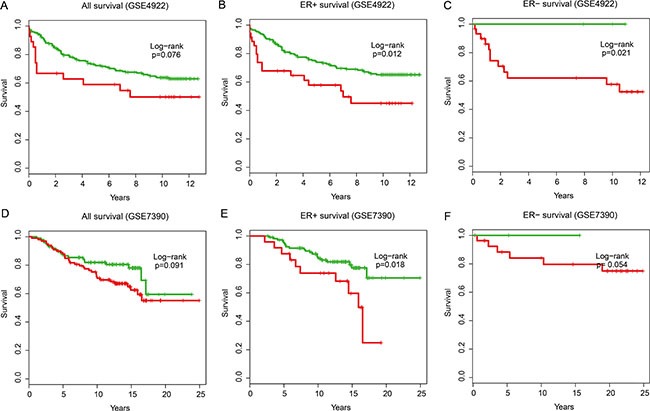
The overall survival analysis in independent validation datasets Kaplan–Meier survival curves were plotted for (**A**) GSE4922 all patients (*n* = 289), (**B**) GSE4922 ER+ patients (*n* = 211), (**C**) GSE4922 ER– patients (*n* = 34), (**D**) GSE7390 all patients (*n* = 198), (**E**) GSE7390 ER+ patients (*n* = 134) and (**F**) GSE7390 ER– patients (*n* = 64).

To confirm the prognostic value of the IgSF module, we validated the 21 gene signatures in four independent microarray datasets (Table 1). Using the same risk score formula, we classified patients in GSE4922 into high-risk and low-risk groups using the median score of the test series as the cutoff point. Consistent with our previous findings, patients in the high-risk group had significantly shorter median OS than those in the low-risk group (Figure 6A, all samples *P* = 0.076; Figure 6B, ER+ samples *P* = 0.012; Figure 6C, ER– samples *P* = 0.021). Similarly, we classified the patients in GSE7390 into a high- and a low-risk group (Figure 6D, all patients, *P* = 0.091; Figure 6E, ER+ patients, *P* = 0.018 and Figure 6F, ER– patients, *P* = 0.054) and obtained similar results.

**Table 1:** The independent microarray datasets used in this study

Datasets	Platform	Number of patients	Overall type	Number of ER+ patients	Number of ER– patients
GSE4922	HG-U133A	289	OS	211	34
GSE7390	HG-U133A	198	OS&DMFS	134	64

Note: Abbreviations: OS, Overall Survival; DMFS, Distant Metastasis-Free Survival.

**Figure 7:**
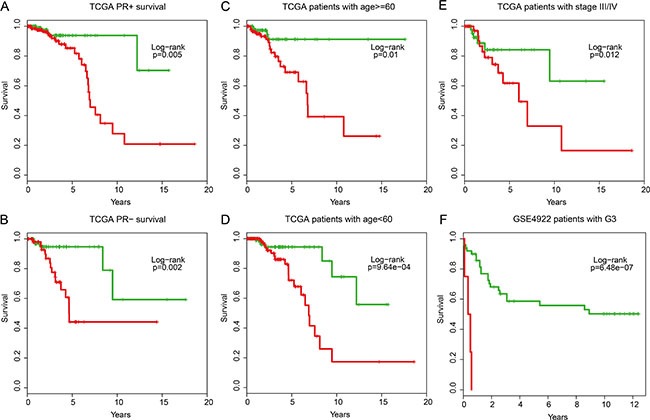
Stratification analyses of the IgSF mutated module with available PR, age, tumor stage and grade information for all patients (**A**) Kaplan-Meier survival curves for TCGA patients with PR+, (**B**) Kaplan-Meier survival curves for TCGA patients with PR–. (**C**) Kaplan-Meier survival curves for TCGA patients with age > = 60, (**D**) Kaplan-Meier survival curves for TCGA patients with age < 60. (**E**) Kaplan-Meier survival curves for TCGA patients with stage III/IV. (**F**) Kaplan-Meier survival curves for GSE4922 patients with G3 grade. *P*-values were calculated using the log-rank test.

After further adjusting for other markers, the univariate analysis indicated that the IgSF module, as an independent risk factor, was significantly associated with the overall survival of breast cancer patients from TCGA (HR = 2.71, 95% CI: 2.02–3.64, *P* = 2.75e-11), GSE4922 (HR = 1.69, 95% CI: 0.94–3.05, *P* = 0.079), and GSE7390 (HR = 1.58, 95% CI: 0.92–2.73, *P* = 0.094) datasets (Table 2). Further, when multivariate analysis was performed to investigate the independence of the module to other clinical factors, the high- and low-risk groups remained independent of other clinical factors in TCGA patients (HR = 2.60, 95% CI: 1.84–3.67, *P* = 4.32e-8), GSE4922 (HR = 1.86, 95% CI: 1.01–3.43, *P* = 0.045), and GSE7390 (HR = 1.70, 95% CI: 0.97–2.96, *p* = 0.060) datasets (Table 2). Data stratification analysis on TCGA patients also indicated that the module was independent of PR, age and tumor stage. It performed similarly in PR category (log-rank test *p* = 0.005 for PR+ group and log-rank test *p* = 0.002 for PR- group) as well as the age category older or younger than 60 (log-rank test *p* = 0.01 for the older patients and log-rank test *P* = 9.64e-4 for the younger patients) and tumor stage III/IV category (log-rank test *P* = 0.012) (Figure 7A–7E). Similar trend was observed in GSE4922 patients with G3 grade (log-rank test *P* = 6.48e-07; Figure 7F). Based on all these data, we conclude that the IgSF-related module is a strong prognostic indicator for breast cancer.

**Table 2:** Statistical analysis of the IgSF module gene signature and overall survival of breast cancer patients in the TCGA and GEO cohorts

Variables	Univariable model			Multivariable model		
	HR	95% CI of HR	*P*-value	HR	95% CI of HR	*P*-value
TCGA (*N* = 450)						
Module risk score	2.7183	2.0252–3.6485	< 0.0001	2.6063	1.8499–3.6720	< 0.0001
ER	1.1332	0.5728–2.2418	0.7195	0.6366	0.2439–1.6615	0.3562
PR	0.7344	0.4214–1.2799	0.2761	0.3486	0.1353–0.8984	0.0291
HER2	0.9361	0.5859–1.4954	0.7823	0.7739	0.4528–1.3227	0.3486
Age	1.0275	1.0044–1.0511	0.0194	1.0529	1.0193–1.0876	0.0018
Stage						
II	2.1360	0.7406–6.1609	0.1602	1.6061	0.5178–4.9821	0.4120
III/IV	3.5429	1.1950–10.5034	0.0225	3.5199	1.0733–11.5434	0.0378
GSE4922 (*N* = 289)						
Module risk score	1.6933	0.9400–3.0505	0.0795	1.8644	1.0133–3.4306	0.0453
ER	0.8583	0.4667–1.5785	0.6230	1.1735	0.6184–2.2271	0.6245
Age	0.9971	0.9815–1.0130	0.7223	0.9999	0.9847–1.0152	0.9862
Grade						
G2	1.8232	1.0336–3.2160	0.0381	1.6455	0.9269–2.9211	0.0890
G3	3.1634	1.6929–5.9113	0.0003	3.2902	1.7080–6.3381	0.0004
GSE7390 (*N* = 226)						
Module risk score	1.5873	0.9234–2.7285	0.0946	1.7023	0.9762–2.9686	0.0608
ER	0.4755	0.2806–0.8060	0.0058	0.4239	0.2277–0.7891	0.0068
Age	1.0131	0.9771–1.0505	0.4802	1.0110	0.9752–1.0482	0.5516
Grade						
G2	1.1056	0.4738–2.5799	0.8165	1.0323	0.4392–2.4266	0.9419
G3	1.3785	0.5976–3.1799	0.4516	0.9614	0.3795–2.4353	0.9338

## DISCUSSION

Elucidating the functional significance and molecular mechanism of IgSF members in breast cancer may provide new opportunities for the early detection and treatment of cancer. A variety of molecular biomarkers in breast cancer have been previously identified. Van de Vijver and others categorized breast cancer based on the gene expression profile of 70 genes and found that their classification was better than standard systems that were based on clinical and histological criteria [32] . Pawitan and others developed a 64-gene signature to predict the response of breast cancer patients to therapy [33]. Venet and others showed that many random gene expression signatures are significantly associated with breast cancer survival, although the underlying mechanism of the biomarkers was unclear implying the need for more effective biomarkers [34]. We performed a global analysis of the IgSF genes in breast cancer and developed a network-based strategy to identify the IgSF-related PPI network and modules. Our study shows that the IgSF network genes closely interact with the cancer driver genes in the mutations enriched module and can serve as a survival-associated biomarker for breast cancer. Also, the IgSF genes participate in the dysregulation of breast cancer driver genes. These results provide a novel understanding of the role of IgSF network in breast cancer progression. Our study also highlighted the importance of immune-related IgSF genes. Immune genes play a key role in cell-cell communication, and dysfunctional immune response cause various diseases in humans including cancers [9]. In breast cancer, the immune genes may modulate the communication between malignant cells and normal cells. We had postulated that the IgSF-related modules mediate breast cancer progression by regulating cancer metastasis. However, functional enrichment analysis showed that the IgSF-related modules were involved in a variety of biological events, including immune response, cell adhesion, biological adhesion and regulation of alpha-beta T cell proliferation. This suggests that in the early stage of breast cancer, IgSF-related modules restrict cell proliferation by regulating signal transduction and the immune response.

In summary, our data shows that the IgSF-related module that is of prognostic importance. Our data also reveal novel functional insights for dysregulated IgSF-related members and modules in the etiology of breast cancer.

## MATERIALS AND METHODS

### Human IgSF gene dataset

The IgSF gene data was downloaded from the HUGO Gene Nomenclature Committee website (HGNC, http://www.genenames.org/), constituting 478 IgSF genes from 651 records, including 42 C1-set, 40 C2-set, 245 Immunoglobulin-like, 161 I-set and 163 V-set domain containing records.

### Breast cancer driver gene dataset

We downloaded cancer associated genes from the Catalogue of Somatic Mutations in Cancer (COSMIC v70; Aug 2014), which is the world's largest and most comprehensive resource for exploring the impact of somatic mutations in human cancer. Firstly, we obtained the breast cancer associated genes from the Cancer Gene Census by searching the keyword “breast” in the tumor types. In addition, we searched cosmic mutation data with the keyword “breast” in the primary site, “y” if the entire genome/exome is sequenced and “CANCER” if the mutations affected the tumor generation in pathology. In summary, we obtained 1,307 unique genes acting as the breast cancer driver genes for subsequent analyses.

### Human protein-protein interaction data

The protein-protein interaction (PPI) data was downloaded from the Human Protein Reference Database (HPRD Release9, http://www.hprd.org/) [35]. It contained more than 42,000 manually curated interactions between 9,826 human genes.

### Analysis of breast cancer gene expression and somatic mutations in clinical datasets

In this study, we focused only on invasive ductal carcinoma (IDC) patients with breast cancer. The breast cancer gene expression, somatic mutation data and the corresponding clinical data was downloaded from The Cancer Genome Atlas (TCGA, http://cancergenome.nih.gov/). The gene expression profiling from 591 breast cancer patients was generated by the UNC__AgilentG4502A_07_3 microarray platform. The samples that contained only expression profiling without clinical data were removed from the analysis. We generated 450 IDC samples that had both expression profiling and clinical data, involving 17,814 genes. Also, the patients were further stratified into the estrogen receptor negative (ER−) and positive (ER+) groups.

### Validation of microarray datasets

The four independent microarray breast cancer datasets [36–39] that were used in this study were obtained from the Gene Expression Omnibus (GEO) database. All the datasets were produced by the HGU133A or HGU133-PLUS2 platform. The datasets were chosen based on the criteria of no less than 100 samples and the availability of clinical outcome data. Raw microarray datasets were normalized using Robust Multichip Average [40]. The three main steps were as follows: background correction, quantile-normalization and log2-transformation. All the probes were mapped based on their EntrezGeneID. When multiple probes were mapped to the same gene ID, the mean value was used to represent the expression value of the single gene. To account for differences in systematic measurement between different datasets, each dataset was standardized independently by transforming the expression of each gene into a mean of 0 and a standard deviation of 1.

### Network construction and analysis of topological features

We constructed a human PPI network based on the HPRD data. Then, we distinguished all the IgSF genes in this network using HGNC IgSF family data. Finally, using IgSF genes and their direct interacting genes in the network (referred to as IgSF neighbor genes), we constructed a sub-network of the human PPI network named the IgSF-directed neighbor network (IDNN). Furthermore, we extracted all the IgSF genes and the breast cancer driver genes to construct an IgSF-directed driver network (IDDN) for subsequent analysis. The Cytoscape software was used for the construction of networks. The topological properties of the IgSF genes were analyzed in both IDNN and IDDN.

### Identifying functional modules in the IgSF networks

Based on the IDDN constructed by the IgSF and breast cancer driver genes, we identified all the network modules using GraphWeb [41], a web server for identifying the network-based biomarkers that best represent the property of the network. The GraphWeb web server was made of three component processes: (i) Network datasets (to input human protein-protein interaction pairs; IDDN in this study), (ii) Network algorithm (we used the betweenness centrality clustering method and the default values were set) and (iii) Network settings (including default edge settings, node setting and module settings with less than 3 nodes and insignificant modules hidden).

### Statistical analysis

Hypergeometric test was used to explore the overlap between the genes in the IgSF-related modules and the top mutated genes in the sub-network for breast cancer. We also studied the enrichment of breast cancer driver genes in IgSF by using this method. Univariate and multivariate analyses were performed using Cox proportional hazards regression model to determine whether the IgSF-related prognostic module was independent of other clinical variables, and adjusted for ER, PR, HER2, age, stage and grade. Hazard ratio (HR) and 95% confidence intervals (CI) were estimated by Cox proportional hazards regression model. To verify if the modules we identified are associated with patient survival, we determined the regression coefficient of every gene in the module related to patient survival using the gene expression data. The classifier was built as a linear combination of the gene expression values of select immune-related genes with the standardized Cox regression coefficient as the weight. A risk score formula for each patient was established by including the expression values of each selected gene, weighed by their estimated regression coefficients in the multivariate Cox regression analysis [42]. Finally, the patients were divided into high- and low-risk groups using the median of the risk score as the threshold. The patients with high-risk scores were classified as poor outcomes. Kaplan-Meier survival plots and log-rank tests by R package “survival” were used to assess the differences in overall survival (OS) time between the high- and low-risk patients. Bioinformatic analysis was performed with R 3.0.0 statistical software.

### Functional enrichment analysis

Functional enrichment analysis at the GO and KEGG levels were performed using DAVID bioinformatics resources (http://david.abcc.ncifcrf.gov/, version 6.7) [43]. The DAVID enrichment analysis was limited to KEGG pathways and GO-FAT biological process (BP) terms with the whole human genome as background. Functional categories with a *p*-value of < 0.05 were considered statistically significant.

## SUPPLEMENTARY MATERIALS FIGURES AND TABLES


