# Supplementary material for: Immunoglobulin superfamily genes are novel prognostic biomarkers for breast cancer
Source: Oncotarget. 2016 Nov 29;8(2):2444–56. doi: 10.18632/oncotarget.13683 (PMC5356814; doi:10.18632/oncotarget.13683)
Supplement: Supplementary file 1 [file oncotarget-08-2444-s001.pdf]

# Immunoglobulin superfamily genes are novel prognostic biomarkers for breast cancer

## Supplementary Materials

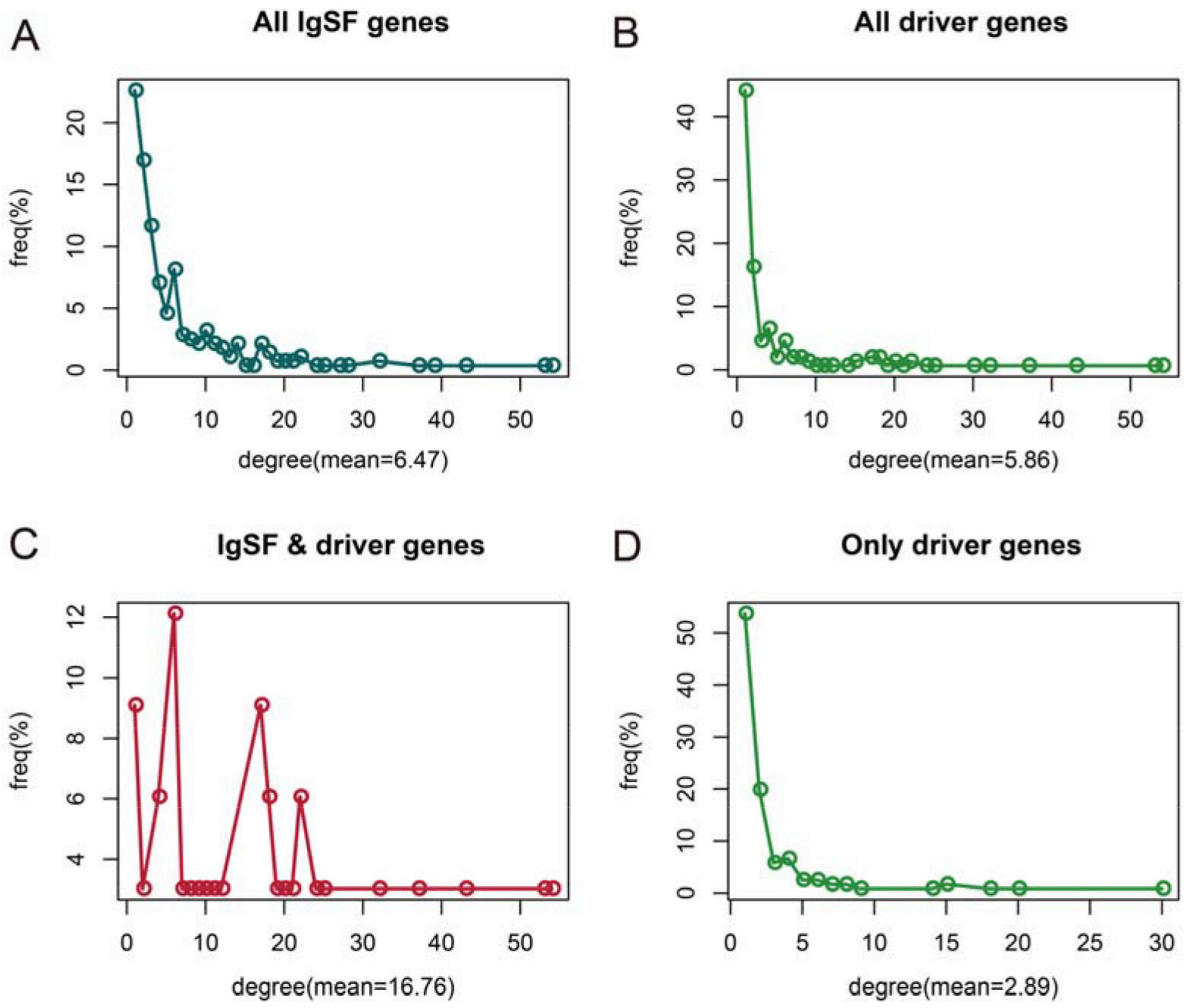


**Supplementary Figure S1: The topology characteristics of IDNN.** (**A**) Degree distribution of all IgSF genes in IDNN, (**B**) Degree distribution of all driver genes in IDNN, (**C**) Degree distribution of the genes belonging to both IgSF and driver gene lists, (**D**) Degree distribution of only the driver genes.

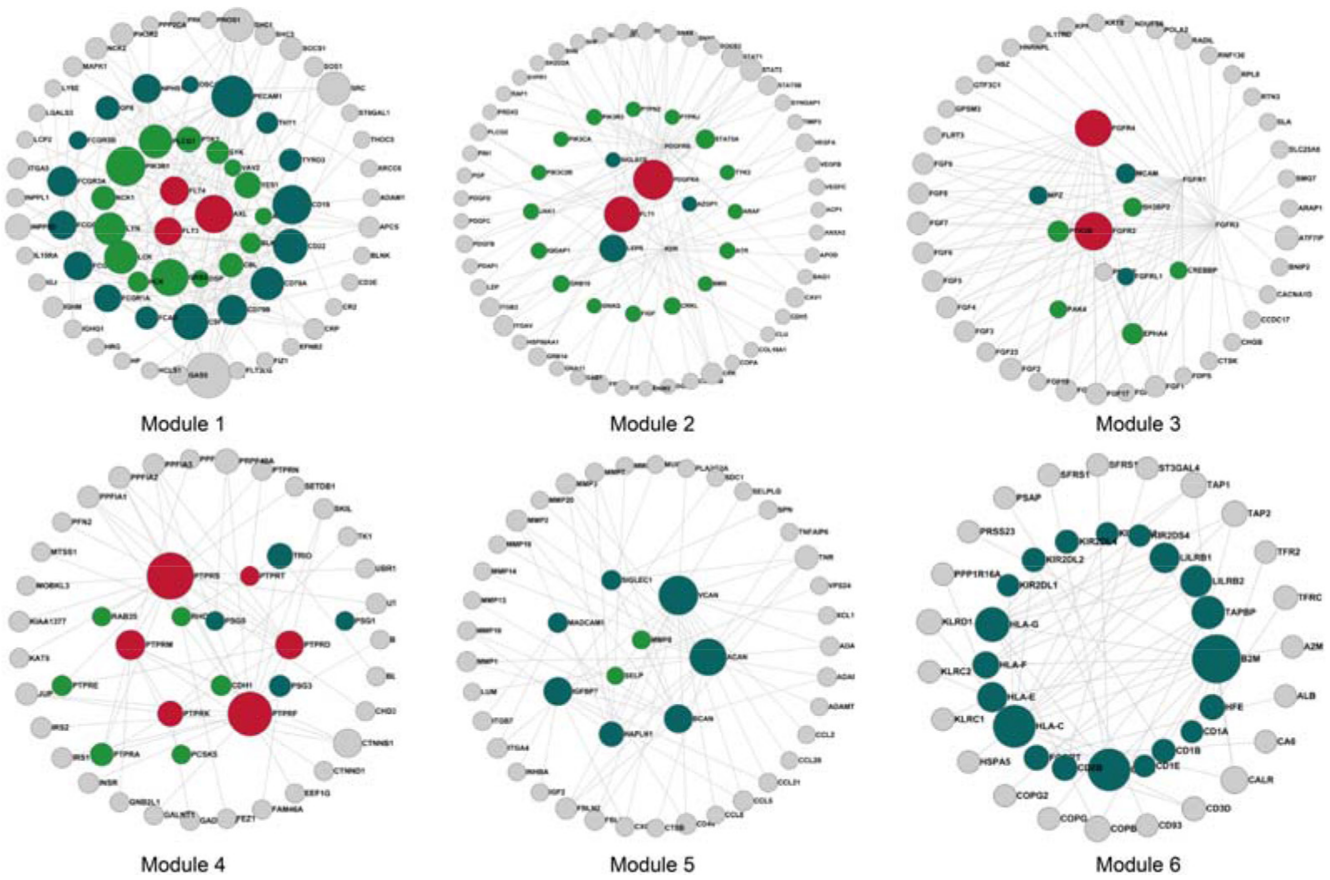


**Supplementary Figure S2: The six high ranked modules obtained from IDNN analyzed by GraphWeb server.**

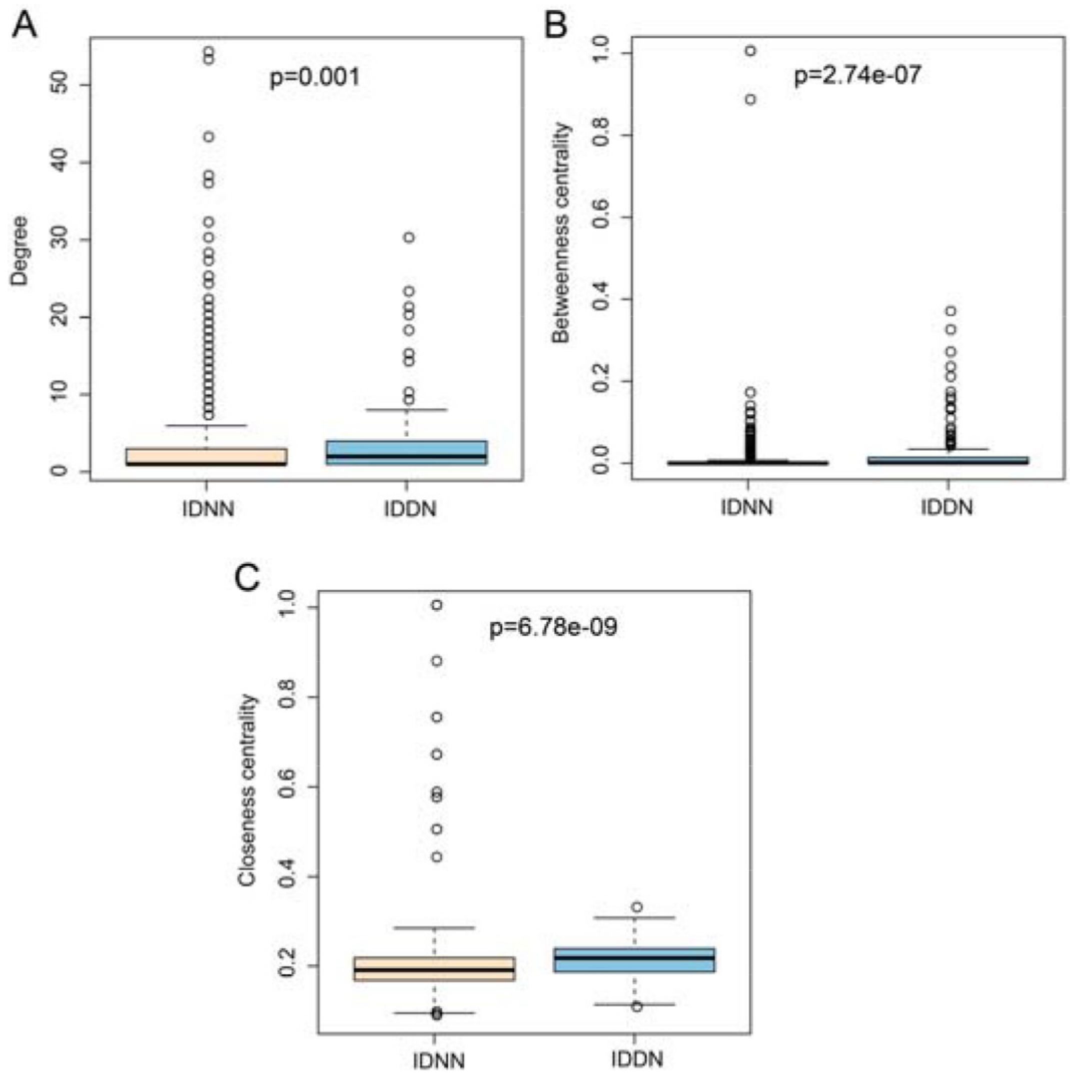


**Supplementary Figure S3: The comparison of topology characteristics between IDNN and IDDN.** (**A**) The IDDN nodes show a higher degree than IDNN nodes. (**B**) The IDDN nodes show higher betweenness centrality than IDNN nodes. (**C**) The IDDN nodes show a higher clustering coefficient than IDNN nodes. *P*-values were calculated based on Wilcoxon rank sum test.

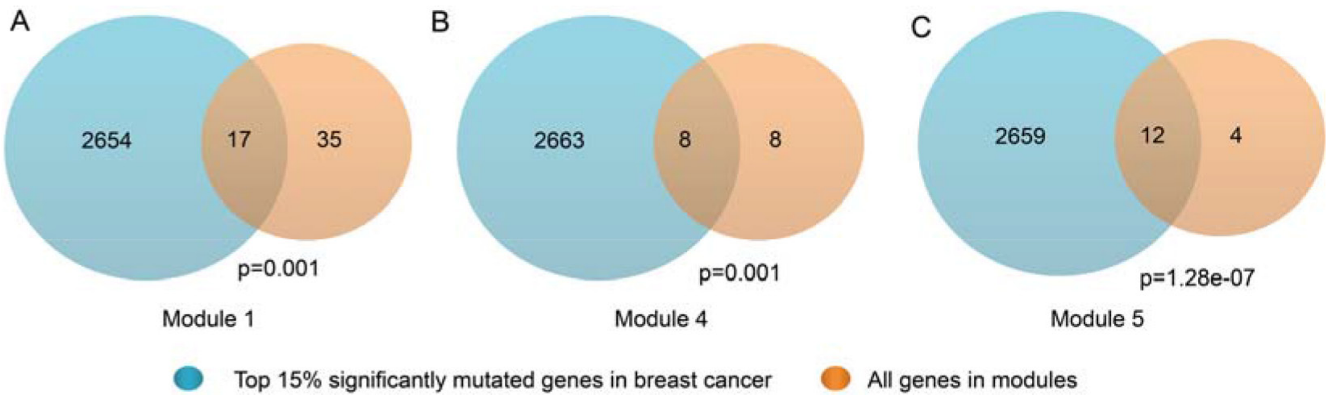


**Supplementary Figure S4: The IgSF-associated modules were enriched with significantly mutated genes in breast cancer.** (**A**) A Venn plot comparing the genes in IgSF-associated module 1 and the top 15% mutated genes in breast cancer. (**B**) A Venn plot comparing genes in the IgSF-associated module 4 and the top 15% mutated genes in breast cancer. (**C**) A Venn plot comparing genes in the IgSF-associated module 5 and the top 15% mutated genes in breast cancer.

**Supplementary Table S1: The IgSF-directed neighbor network.** See Supplementary_Table_S1

**Supplementary Table S2: The 250 IgSF genes associated with breast cancer driver genes in IDNN.** See Supplementary_Table_S2

**Supplementary Table S3: Breast cancer driver genes in six IDNN modules**

| | n | m | k | x | *p*-value |
|---|---|---|---|---|---|
| IDNN | 1050 | 283 | 154 | 33 | 0.998 |
| Module1 | 74 | 20 | 18 | 3 | 0.930 |
| Module2 | 72 | 7 | 20 | 4 | 0.088 |
| Module3 | 52 | 7 | 8 | 3 | 0.064 |
| Module4 | 47 | 10 | 12 | 6 | 0.010 |
| Module5 | 48 | 7 | 2 | 0 | 1.000 |
| Module6 | 23 | 20 | 0 | 0 | 1.000 |

Note: n, the number of the nodes in network or module; m, the number of IgSF genes; k, the number of breast cancer driver genes; x, the number of the intersection of driver genes and IgSF.

**Supplementary Table S4: The IgSF-directed driver network.** See Supplementary_Table_S4

## Supplementary Table S5: The top 5 Go terms for lgSF module genes in breast cancer

| Rank | Biological process (module 1) | FDR |
|---|---|---|
| 1 | GO:0007169~transmembrane RTK signaling pathway | 7.54E–17 |
| 2 | GO:0007167~enzyme receptor protein signaling pathway | 2.33E–16 |
| 3 | GO:0006468~protein amino acid phosphorylation | 2.69E–14 |
| 4 | GO:0016310~phosphorylation | 1.78E–13 |
| 5 | GO:0006796~phosphate metabolic process | 8.65E–12 |
| Rank | Biological process (module 2) | *P* Value |
| 1 | GO:0006955~immune response | 0.0058 |
| 2 | GO:0007155~cell adhesion | 0.0061 |
| 3 | GO:0022610~biological adhesion | 0.0062 |
| 4 | GO:0046640~regulation of alpha–beta T cell proliferation | 0.0143 |
| 5 | GO:0007169~transmembrane receptor protein tyrosine kinase signaling pathway | 0.0249 |
| Rank | Biological process (module 3) | FDR |
| 1 | GO:0042110~T cell activation | 4.77E–13 |
| 2 | GO:0046649~lymphocyte activation | 4.47E–11 |
| 3 | GO:0045321~leukocyte activation | 3.22E–10 |
| 4 | GO:0001775~cell activation | 1.77E–09 |
| 5 | GO:0002696~positively regulate leukocyte activation | 9.96E–08 |
| Rank | Biological process (module 4) | FDR |
| 1 | GO:0006470~protein amino acid dephosphorylation | 5.28E–06 |
| 2 | GO:0016311~dephosphorylation | 1.28E–05 |
| 3 | GO:0007185~transmembrane RTP signaling pathway | 5.42E–05 |
| 4 | GO:0006793~phosphorus metabolic process | 2.35E–04 |
| 5 | GO:0006796~phosphate metabolic process | 2.35E–04 |
| Rank | Biological process (module 5) | *P* Value |
| 1 | GO:0006936~muscle contraction | 1.04E–05 |
| 2 | GO:0003012~muscle system process | 1.50E–05 |
| 3 | GO:0032989~cellular component morphogenesis | 2.25E–05 |
| 4 | GO:0000902~cell morphogenesis | 2.79E–04 |
| 5 | GO:0002696~positively regulate leukocyte activation | 3.32E–04 |
| Rank | Biological process (module 6) | FDR |
| 1 | GO:0007155~cell adhesion | 2.80E–09 |
| 2 | GO:0022610~biological adhesion | 2.84E–09 |
| 3 | GO:0016337~cell–cell adhesion | 9.83E–08 |
| 4 | GO:0007159~leukocyte adhesion | 1.94E–05 |
| 5 | GO:0022614~membrane to membrane docking | 0.0133 |

**Supplementary Table S6: The SNPs in six immune genes with breast cancer risk mutations**

| Gene | SNP | Genotype | Case/control | OR(95%CI) | *P* |
|---|---|---|---|---|---|
| BTLA | rs2705535(G > A) | GG | 368/367 | 1(reference) | |
| | | AG/AA | 196/139 | 0.711(0.543–0.931) | 1.21E–02 |
| ITGAL | rs11574944(C > T) | CC | 374/1 | 1(reference) | |
| | | CT/TT | 162/562 | 1317.517(224.857–4.503E+15) | 1.63E–161 |
| | rs2230433(G > C) | GG | 290/391 | 1(reference) | |
| | | CG/CC | 247/197 | 0.592(0.461–0.758) | 1.92E–05 |
| | rs8058823(A > G) | AA | 519/576 | 1(reference) | |
| | | AG | 14/1 | 0.064(0.002–0.427) | 3.15E–04 |
| CTLA4 | rs4553808(A > G) | AA | 403/425 | 1(reference) | |
| | | AG/GG | 169/126 | 0.707(0.536–0.932) | 1.21E–02 |
| ICOS | rs10932029(T > C) | TT | 418/599 | 1(reference) | |
| | | CT/CC | 67/66 | 0.688(0.471–1.005) | 4.96E–02 |
| PDCD1 | rs2227981(T > C) | TT/CT | 191/234 | 1(reference) | |
| | | CC | 294/244 | 0.678(0.520–0.882) | 2.86E–03 |
| VTCN1 | rs10754339(G > A) | GG/AG | 223/180 | 1(reference) | |
| | | AA | 276/324 | 1.454(1.119–1.890) | 3.80E–03 |
| | rs10801935(C > A) | CC | 8/24 | 1(reference) | |
| | | AC/AA | 491/480 | 0.326(0.125–0.760) | 6.17E–03 |
| | rs3738414(G > A) | GG | 324/278 | 1(reference) | |
| | | AG/AA | 175/226 | 1.504(1.158–1.957) | 1.61E–03 |

**Supplementary Table S7: The Cox regression coeffcients of the IgSF module genes**

| Gene | Total patients | ER+ patients | ER- patients |
|---|---|---|---|
| BTLA | 0.1843 | 0.0919 | −0.1358 |
| CD33 | −0.2236 | −0.0311 | −0.0313 |
| FCRL3 | −0.3445 | −0.2253 | −0.3128 |
| FGR | −1.8167 | −1.0064 | 1.7695 |
| LAIR1 | 1.2731 | 1.1233 | 2.1443 |
| LEPR | 0.6172 | 0.3979 | 1.4992 |
| LILRB4 | −0.9200 | −1.3209 | −1.5106 |
| MPZL1 | −0.0723 | −0.4074 | −0.5595 |
| PILRA | 0.2285 | 0.2211 | −1.3978 |
| PILRB | 0.0211 | 0.0002 | 1.6779 |
| PTPN11 | 1.1016 | 0.6804 | 2.0602 |
| SIGLEC11 | 0.3977 | 0.0441 | 1.3695 |
| SIGLEC12 | 0.5218 | 0.7125 | −0.1716 |
| SIGLEC7 | −0.1240 | −0.2758 | −0.0118 |
| SIRPA | −0.5044 | −0.3737 | 0.3692 |
| SLAMF1 | 0.4980 | 0.3466 | −0.5214 |
| SLAMF6 | 0.1499 | 0.2377 | −1.3167 |
| TREML1 | 2.2445 | 1.6066 | 5.1610 |
| TRIM2 | −0.2165 | −0.1874 | −0.3685 |
| ZAP70 | −1.1718 | −1.1351 | −1.4752 |
